# Unravelling Pea–Ascochyta Blight Interaction and Its Implications for Pea Breeding

**DOI:** 10.3390/ijms27104174

**Published:** 2026-05-08

**Authors:** Manuel Alejandro Jiménez-Vaquero, Diego Rubiales

**Affiliations:** 1 Institute for Sustainable Agriculture, Spanish National Research Council (IAS-CSIC), 14004 Córdoba, Spain; diego.rubiales@ias.csic.es; 2 Programa de Doctorado de Ingeniería Agraria, Alimentaria, Forestal y de Desarrollo Rural Sostenible, Universidad de Córdoba, 14071 Córdoba, Spain

**Keywords:** *Pisum sativum*, Ascochyta blight, quantitative resistance, phenotyping, omics, multi-omics, molecular breeding, marker-assisted selection, genomic selection

## Abstract

Pea (*Pisum sativum* L.) is an important temperate grain legume crop of high nutritional and agronomic value. Ascochyta blight, caused by a multi-species complex of necrotrophic fungi, remains a major constraint for pea production worldwide. This review synthesizes the available genetic, physiological and molecular knowledge on the pea–Ascochyta blight pathosystem, with emphasis on the genetic architecture of resistance, host defense mechanisms and the recent contributions from the omics disciplines. Current evidence indicates that genetic resistance to the various Ascochyta blight pathogens is incomplete and multicomponent, being associated with loci of small to moderate effect, with expression depending on organ, developmental stage and environment. Under field conditions, the observed phenotypes reflect the interaction between physiological resistance, plant architecture, phenology, canopy microenvironment and epidemic dynamics. Together, these factors bias phenotyping and limit the transferability of molecular markers. The practical value of these markers for use in marker-assisted selection (MAS) and genomic selection (GS) is presented and critically discussed. Future progress in breeding for Ascochyta blight resistance will depend on integrating molecular knowledge with a careful definition of ideotypes, well-calibrated phenotyping and multi-environment validation.

## 1. Introduction

Pea (*Pisum sativum* L.) is a legume crop that provides a valuable source of protein and other nutrients in human and animal food chains. Depending on their end use, pea cultivars are classified into three main groups, including green/garden pea (intended for consumption in the fresh state), field/dry pea (for dry grain production, used mainly for feed), and forage pea [1]. Pea is widely grown as a cool-season crop in temperate regions worldwide, and its cultivation is of substantial economic importance in several countries [2]. The Russian Federation and Canada are the main producers of field pea, whereas China and India are the main producers of garden pea, based on annual net production. At the global scale, average production over the 2020–2024 seasons was 13.9 million tonnes per year for field pea, grown on 7.3 million ha, and 21.5 million tonnes per year for garden pea, grown on 2.7 million ha [3].

Like other legumes, pea has a high agronomic value. Its role in crop rotations is particularly relevant due to its capacity to fix atmospheric nitrogen. Thus, it contributes to the nutrient balance of soils, reducing the reliance on synthetic nitrogen fertilizers. In low-input farming, this is crucial in terms of production. Accordingly, the promotion and expansion of legume cultivation, like pea, is a milestone for sustainable farming systems [4].

Therefore, it is necessary to address the factors that limit pea production. Among these constraints, biotic stresses are of great concern. These include fungal, bacterial and viral diseases, pests, and parasitic weeds. In particular, Ascochyta blight is a major constraint for pea production worldwide. In the literature, the disease syndrome is also referred to by regional names such as blackspot/black spot (Australia) or Mycosphaerella blight (North America). Disease pressure leads to yield reduction by decreasing green leaf area, assimilate supply and yield components, with consequent effects on partitioning efficiency [5]. In seasons conducive to epidemics, infection compromises yield stability and seed quality, leading to yield losses of ~25–75% depending on cultivar susceptibility and environmental conditions [6,7]. During cool to mild, wet periods, recurrent rainfall and prolonged canopy wetness favor infection, lesion expansion and spore release [8]. Pea Ascochyta blight is caused by a complex of closely related necrotrophic fungal species that can cause disease alone or in combination. Such heterogeneity in the pathogenic challenge complicates the implementation of management strategies and their extrapolation across environments and seasons [9].

Ascochyta blight control is usually framed as management of epidemic risk. Current protection programs rely on combinations of agronomic practices, such as sowing calendars designed to modulate the timing of exposure to infection and its spread [6]. Fungicide treatments are not cost-effective in commercial plots, as they provide incomplete control that is insufficient when epidemic pressure is high [8]. For this reason, host genetic resistance is considered the most important component of integrated management. An effective deployment of host resistance brings the added value of reducing reliance on chemical fungicides [10]. However, resistance in current elite cultivars is typically partial, and its expression depends strongly on epidemic context [8].

Under field conditions, heritable physiological resistance to Ascochyta blight coexists with other architectural and phenological features of the crop that modulate exposure to the pathogen and disease development [11,12]. The composite nature of this trait highlights the importance of examining the genotype-by-environment interaction on resistant germplasm. Moreover, it also makes it difficult to compare results across trials, seasons and experimental approaches [13]. Over the last decades, the research community has generated a substantial volume of advances in pea resistance to Ascochyta blight, providing genetic markers, molecular-scale knowledge of the pathosystem, and omics resources [14]. At this point, it is relevant to examine if this evidence holds under real cropping conditions, and how it can be translated into management decisions with agronomic relevance and breeding tools.

This review covers the available genetic and molecular evidence for the pea–Ascochyta blight pathosystem and discusses its implications and utility for breeding resistance in pea. It also provides a critical synthesis of contributions from recent omics disciplines, assessing the extent to which they have translated into effective breeding strategies. Finally, it addresses intrinsic features of the Ascochyta blight resistance trait that can bias or constrain the success of breeding pea for resistance.

## 2. The Pea–Ascochyta Blight Pathosystem

### 2.1. Ascochyta Blight as a Multi-Species Complex

In pea, Ascochyta blight is caused by multiple phylogenetically related species of necrotrophic fungi. Therefore, under natural infection, the observed response of the pea to disease may depend on the local composition of the complex [8,9,15]. Within the disease complex, *Ascochyta pisi*, *Didymella pinodes* and *Didymella pinodella* have been historically considered the main Ascochyta blight members. Out of these, *D. pinodes* appears to be the most aggressive and economically important [8,16,17,18]. In Australia, *Ascochyta koolunga* is a common causal agent, and other minor species have occasionally been reported [19,20,21]. Care is therefore needed to monitor the species present in the area and to prevent the introduction of new pathogenic species [21,22,23].

Taxonomy within Didymellaceae has been revised repeatedly. For this reason, the literature often retains historical taxonomic assignments that are no longer valid. As shown in Table 1, these pathogens have been referred to under different names in earlier studies, reflecting repeated taxonomic revisions within Didymellaceae [24,25,26,27,28]. For consistency, we use the current taxonomy in this review: *A. pisi*, *A. koolunga*, *D. pinodes* and *D. pinodella* [29,30,31,32]. Throughout the text, we specify the pathogenic species to which the reported information refers. In contrast, for those cases where the original source did not specify the pathogen identity, we employ the general term “Ascochyta blight”.

### 2.2. Symptomatology and Epidemiology

**Figure 1:**
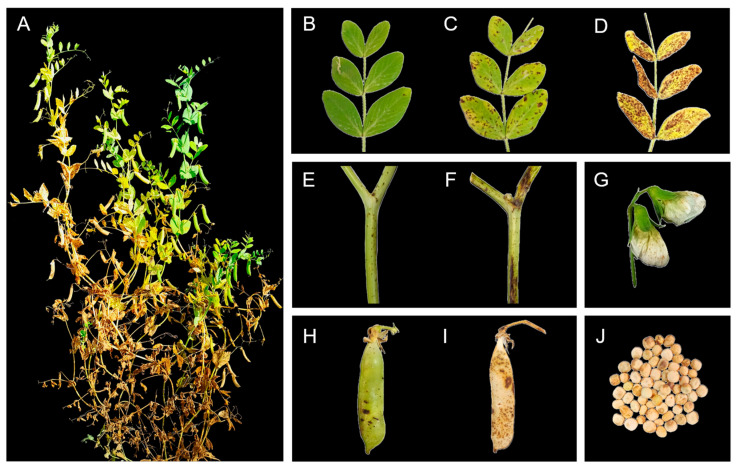
Typical symptoms caused by Ascochyta blight pathogens in *Pisum sativum* L. Representative symptoms observed in pea plants under field conditions: whole plant showing disease development across the canopy (**A**); leaf symptoms at early, intermediate, and advanced stages of lesion development (**B**–**D**); stem and node infection symptoms at early and advanced stages (**E**,**F**); symptoms on floral structures (**G**); pod symptoms during pod filling and after drying (**H**,**I**); and symptoms on mature seeds (**J**).

In pea, Ascochyta blight is expressed as a spectrum of necrotic symptoms in the susceptible organs of the plant (Figure 1). In leaves, lesions often initiate as small, irregular flecks that expand under favorable environmental conditions, becoming darker and coalescing into larger necrotic areas that reduce functional photosynthetic surface and accelerate organ senescence [17,25]. Under severe epidemics, infected leaves may desiccate [7]. As lesions mature, pycnidia (i.e., conidiomata) are typically formed in the necrotic center of the lesions (although alternative patterns of pycnidial distribution within lesions have been described). These structures act as sources of conidia (i.e., pycnidiospores) that constitute the agents of asexual reproduction and spread of the fungus [7]. Severe infection in flowers can surround and girdle the sepal, causing flower drop or abortion of young pods. Pod infection is particularly relevant because it links the foliar syndrome with seed infection, deteriorating seed quality, and contributing to the persistence of the epidemic across seasons. Pod infections established before the beginning of seed filling have a greater impact on seed quality [34]. Infected seeds have a discolored or tanned appearance, with abnormal and irregular shape and size. Pod lesions are elongated spots that develop into oval, sunken areas with dark margins [7].

In stems, symptoms often appear as discrete lesions that extend into purple to dark, elongated streaks, most evident around nodes. Coalescence of these lesions can lead to partial or complete girdling of the stem, promoting its premature senescence or loss of functional integrity [25]. Ascochyta blight can also present as foot rot syndrome at the stem base, with a darkening appearance that can extend to the crown and upper root. *D. pinodella* is the pathogen most frequently associated with this syndrome [9,17,35,36,37]. In seedlings, discoloration can appear in the hypocotyl, cotyledons and taproot. Severe infections can lead to seedling death [7].

Once infection is established, primary lesions develop on susceptible plants. If microenvironmental conditions are favorable, pycnidia are formed and conidia are released as secondary inoculum. These conidia are locally dispersed by splash of rain or dew droplets, resulting in the polycyclic epidemic typical of humid climates. Disease progression commonly occurs upwards through the plant canopy. Basal leaves and stems are infected first, and disease subsequently spreads towards new foci at the upper nodes [8].

Conidia can germinate across a wide thermal range (around 4–35 °C, with an optimum of ~28 °C), but infection, lesion expansion, and pycnidial production are maximized around 20 °C. At suboptimal temperatures, longer periods of leaf wetness are required to sustain disease development. Under favorable conditions and frequent rainfall, disease progression matches the rate of plant growth, whereas during dry periods, canopy growth can temporarily outpace pathogen spread. The largest increase in disease development commonly occurs during the reproductive window of the crop [7,8,38].

### 2.3. Field Management of Ascochyta Blight Disease

Control of pea Ascochyta blight has relied on a limited set of agronomic and chemical measures aimed at reducing inoculum carryover and modulating crop exposure over the season. Their impact is typically context-dependent, particularly sensitive to the climatic conditions of each growing season [8].

The initial Ascochyta blight inoculum can originate from seed, soil and, in particular, infected crop residues from previous seasons. For *D. pinodes*, pseudothecia (i.e., ascostromata) present in stubble residues release infective ascospores, which are then dispersed by wind or rain-splash, providing primary inoculum for infection. In addition to pea, *D. pinodes* can moderately infect other species, which may act as local reservoirs for epidemics [15].

The usual cultural practices in this context are crop rotation and crop residue management, as debris can act as an inoculum source for the onset of epidemics [8]. Seed is another important control point: infected seed lots can contribute to epidemic establishment, so seed sanitation control reduces the risk of early infection [8,39,40]. Taken together, these measures tend to lower the probability of initial infection, but they rarely provide sufficient protection on their own in seasons that are climatically favorable for the pathogen.

Other practices that modulate the timing of crop exposure to the pathogen are also important. Sowing date can alter synchrony between crop development and the climatic window most favorable to disease spread. However, delayed sowing often entails agronomic trade-offs [41]. Canopy structure can also influence whether the crop provides a microenvironment that is more or less favorable for Ascochyta blight development and dispersal. It can be shaped by sowing density or cultivar architecture traits [8].

Chemical control is feasible, although not always cost-effective [7]. Fungicides used for Ascochyta blight control on legume crops belong to three different classes, namely succinate dehydrogenase inhibitors (SDHIs), demethylation inhibitors (DMIs), and quinone outside inhibitors (QoIs). The site-specific mode of action (MoA) of these fungicides, together with the polycyclic nature of the disease, with both sexual and asexual reproduction and easy spore dispersal, has raised concerns about the development of fungicide resistance in the fungal populations. The polycyclic nature of the disease predisposes growers to repeat fungicide applications as disease severity can increase rapidly when the weather is favorable [42]. Currently, QoI fungicides have been the most commonly used for pre- and post-infection management of Ascochyta blights in the various legume crops [42,43]. Before other site-specific options became widely available, QoIs were a major fungicide group used in pulse crops, and resistance developed rapidly, particularly in *D. rabiei* affecting chickpea [44] but also in *D. pinodes* [43] and *A. pisi* [45]. To contain the spread of QoI resistance, monitoring is key. Other practices to prevent resistance development include restricting the frequency of QoI treatments to two to four applications per season, using QoIs as a preventive rather than a reactive strategy, avoiding sequential applications of QoI products, alternating with other fungicide MoAs when multiple applications are necessary (e.g., SDHIs, DMIs), using pre-mixtures or tank mixtures of products with different MoAs, and always applying the recommended labeled rate [46]. DMI fungicides, such as Prothioconazole, have a broad-spectrum activity on many plant pathogens by inhibiting fungal cell membrane development through interfence with ergosterol biosynthesis. Despite their site-specific mode of action, DMIs fungicides are considered to be at medium risk for fungicide resistance development. Reduced sensitivity of *D. pinodes* to prothioconazole has been reported in vitro [9,47]. SDHIs fungicides, such as Boscalid and Fluxapyroxad, inhibit mitochondrial respiration by targeting succinate dehydrogenase. In a baseline sensitivity study across Ascochyta blight fungi, *D. pinodes* showed low mean EC_50_ (half maximal effective concentration) values for boscalid and to fluxapyroxad, indicating high baseline sensitivity [42].

Nevertheless, given the limited and variable efficacy of individual management measures for Ascochyta blight, host resistance remains the central tool on which additional protection strategies can be built [8,48]. Its value in field management depends on the availability of resistance that is consistent, agronomically useful and robust across relevant disease contexts.

## 3. Assessment of Pea Response to Ascochyta Blight

### 3.1. Disease Phenotyping

#### 3.1.1. Phenotyping Strategies

Approaches and techniques to evaluate Ascochyta blight response in pea have diversified from classical field scoring to controlled assays and image-based assessments. The main goals that drove this transition were the improvement in reproducibility, throughput, and phenotyping depth.

Field phenotyping remains the most widely used approach to characterize responses to Ascochyta blight and has supported both germplasm screenings and genetic mapping studies. Within this framework, an epidemic is commonly allowed to develop under natural conditions [49,50,51].

Whole-plant assays under controlled conditions allow more uniform disease development among individuals. These settings improve evaluation efficiency for large genotype panels that could exceed field handling capacities [22,52,53,54]. Controlled conditions assays are usually carried out on young seedlings, using well-defined inocula. Different protocols have been proposed, all of them including a period of incubation under controlled humidity and temperature after inoculation [22,54,55,56]. Such designs are common in trials where inoculum has a central role, enabling specific comparisons between pathogens or isolates [57].

Detached organ assays (most often involving leaflets and stipules) have become a common tool for characterizing quantitative lesion components under controlled conditions. Recent assay designs have been optimized for higher throughput [58,59]. Detached organ assays maximise scalability, whereas whole-plant assays are preferred when whole-plant response is of interest [60].

Image analysis is increasingly used for plant disease phenotyping, particularly in controlled assays. These methodologies facilitate the assessment of quantitative traits and their dynamics over the course of infection. More importantly, the time required for phenotyping is reduced when applying these methodologies. In pea, this trend has already supported evaluation studies for other biotic stresses, in which digital workflows complement conventional assessments [61,62]. To quantify leaf area stained for specific detection of hydrogen peroxide, Joshi et al. [58] used image analysis on photographs through a pipeline based on ‘ImageJ’ software (version 1.53c). For detached stipules, time series of images have been generated to parameterize Ascochyta blight lesion expansion [63]. These variables improve comparability across standardized protocols and studies, and proved key to achieving throughput gains in recent phenotyping designs [60].

#### 3.1.2. Variables and Scales

Across Ascochyta blight phenotyping systems, response to infection has been quantified using heterogeneous variables, their interpretation being highly context-dependent. A number of experimental factors shape the definition of these scales, including the type of symptom assessed, the time of scoring (relative to epidemic development), the number of scorings, and the number of categories defined, among others. When these features are defined differently across studies, the resulting scales can be difficult to compare.

In field trials, severity can be recorded as a percentage estimate of affected tissue area at the canopy level. This class of variables has an intuitive definition and a direct interpretation as overall phenotype [49]. However, variation between measurements is more evident for continuous scales like these. By contrast, ordinal scales combine multiple components of the phenotype into a set of predefined categories (e.g., lesion frequency and coalescence, total affected area, presence of desiccated tissue). Ordinal scales have been widely used in Ascochyta blight studies. Well-established examples are the 0–9 scale, where 0 denotes no disease, and 9 denotes a completely affected plant [64,65], or the 0–5 scale that compresses symptoms observed across the different plant organs [15,66,67]. When repeated assessments are feasible, data can be integrated into disease-progress variables, with the AUDPC and its related derivatives being the most common [68]. In addition, some studies also report incidence alongside severity, which can be informative when establishment or spread proves heterogeneous across individuals or plots [68].

Controlled assays (whole-plant or detached organ) are more prone to the implementation of continuous variables that improve the depth of information obtained. This includes variables such as lesion area/radius, proportion of necrotic area, and lesion frequency. Detached organ settings are particularly well suited to these assessments and are highly compatible with digital image methodologies [58,63].

### 3.2. Reported Sources of Resistance in Pisum Germplasm

The search for pea genotypes resistant to Ascochyta blight emerged from the need to identify which varieties were most suitable to sow in order to avoid the disease or reduce its impact on yield. For this reason, early work was often closely linked to local breeding programs or variety evaluation trials. Studies developed in Canada during the 1950s and 1960s mainly focused on the response to *A. pisi*. In this context, Lyall and Wallen [69] described the breeding line Ottawa A-100 as a highly resistant source. Gfeller and Wallen [70] later characterized the pea line Creamette (subsequently released as Century) as a useful resistant cultivar, which rapidly became widely grown because of its high level of protection against *A. pisi* [40].

Further screenings for Ascochyta blight resistance in cultivated germplasm focused on other pathogenic species that grew in importance over the years. Ali et al. [71] evaluated a set of pea lines against isolates of *D. pinodes*, *A. pisi* and *D. pinodella* collected in South Australia and described differential responses among species and pathotypes. Other studies compared cultivar reactions to *D. pinodes* [22,52] and *D. pinodella* [35], while early evaluations also highlighted partial resistance in stems and leaves within small germplasm sets [53]. Large-scale screenings in the late 1990s and early 2000s consistently showed that high resistance is uncommon in cultivated pea [72,73]. Kraft et al. [49] screened 2936 accessions from the USDA (United States Department of Agriculture) against *D. pinodes* and found no genotype exceeding the partial resistance of the check cultivar Radley, with only a small subset matching it. Francis et al. [74] screened around 500 lines obtained from the N.I. Vavilov All-Russian Institute of Plant Genetic Resources (VIR) and the International Center for Agricultural Research in the Dry Areas (ICARDA). They reported that around 40 of these lines showed partial field resistance to Ascochyta blight.

Against the lack of fair resistance in *P. sativum* germplasm, screening efforts extended the search to landraces and wild pea relatives. The potential value of related species within the genus *Pisum* as sources of resistance to *D. pinodes* was highlighted by Wroth [75] and supported by later evaluations using multi-species panels [76]. In this context, Fondevilla et al. [77] evaluated the response to *D. pinodes* of 78 *Pisum* spp. and reported that 14 showed high levels of resistance, which proved effective both under controlled conditions and field settings. The strongest resistance levels were observed in *P. fulvum* and in *P. sativum* ssp. *elatius* and ssp. *syriacum*. Subsequent evaluations have continued to support the value of non-cultivated *Pisum* material against *D. pinodes* [50,78]. A comprehensive summary of reported sources of partial resistance to Ascochyta blight pathogens within the *Pisum* genus is provided in Table 2. More recent evaluations have expanded this evidence across broader pathogen panels and have reinforced the need to interpret reported resistance sources in relation to the causal agent assessed [9,57,58,60,79,80].

In screening assays, partially resistant check genotypes are commonly used as reference points, although their value depends on the pathogen species assessed and the experimental context. For partial resistance to *D. pinodes*, the typical reference cultivar is Radley, an old commercial variety developed in the UK, with green seeds, semi-leafless type, and a short growth habit [49,72,78]. CDC Striker has been widely used in Canadian works as a check for moderate resistance in germplasm screenings and introgression studies [78,84]. Less frequently, the cultivars Danto, Majoret and Carneval were used as partially resistant checks in *D. pinodes* inoculations [72,84,85]. For resistance to *A. pisi*, some reference genotypes are specified in the official distinctness test protocols from the Community Plant Variety Office (CPVO). In particular, Rondo has long been prescribed for checking response to *A. pisi* race C, as a standard resistant cultivar. More recent protocols list Madonna and Nina as additional resistant checks [86,87]. In technical documents and breeding catalogues, reference genotypes are used to track genetic improvement over time. This role is commonly assigned to cultivars that are well-established within each region and offer balanced phenotypic profiles. In Australia, varietal guides present Kaspa as a field pea ideotype, with intermediate susceptibility to Ascochyta blight and good agronomic performance [88]. In western Canada, this role has historically relied on Radley in the Field Pea Cooperative Registration Tests [78], whereas CDC Striker is the standard in regional variety catalogues [89,90].

### 3.3. Inoculum Composition as a Determinant of Pea Response to Ascochyta Blight

Given the multi-species nature of the Ascochyta blight complex, it is difficult to compare resistance evidence across studies when the causal agents are different or not clearly defined. In such cases, resistance may appear inconsistent across experimental contexts.

This is routinely reflected in comparative studies evaluating more than one causal agent, which often report contrasts in response to different species. Recently, Annan et al. [60] described materials with a strong response to *A. pisi* that did not translate into resistance against *D. pinodes*. Similarly, Joshi et al. [58] compared responses to *D. pinodella* and *D. pinodes* and observed differential patterns. For this reason, many findings on resistance mechanisms or sources are pathogen-specific and can only be tentatively extended to other members of the Ascochyta blight complex [8,57,58].

Within *A. pisi*, different physiological races have long been recognized [82]. In *D. pinodes*, differential reactions on pea genotypes have also been described across isolates. Some regional studies have grouped *D. pinodes* isolates into pathotypes, indicating pathogenic variation [83,85,91]. For *D. pinodella*, evidence is more limited and mainly supports variation in aggressiveness among isolates [37,58].

Furthermore, competitive and facilitative interactions may occur in co-infections with more than one Ascochyta blight pathogen involved. This leads to resistance or susceptibility responses different from those observed in single infections. Host genetic background has been proposed to modulate these dynamics [7,60,92].

### 3.4. Field Resistance as a Composite Phenotype

Field phenotyping for Ascochyta blight remains conclusive for classifying a resistance trait as deployable. Field settings implicitly test the stability of the genotype response under uncontrolled environmental conditions, anticipating its real performance at commercial scale [8,93]. However, this also makes canopy-level severity a composite phenotype, reflecting the combined effects of partial physiological resistance, microenvironment, crop developmental status, and inoculum dynamics, among other factors [8,17].

In the field, the scope for a given genotype to express its resistance varies across seasons and locations, as pathogen pressure, climatic conditions and local microenvironment jointly determine epidemic development. Schoeny et al. [94] formalized these determinants in a model based on climatic indices, highlighting the climatic profile of each season as highly decisive for the trajectory of the epidemic. Spatial heterogeneity in microclimate within field settings is an additional factor less often highlighted [13,95]. Ascochyta blight intensity frequently shows strong local structure, driven by variations in soil conditions, debris or other inoculum foci distribution, local topography, and moisture-related gradients.

Not all of the sources of variation in field phenotypes are strictly external to the host. On the contrary, several of them are partly mediated by genotype traits that coexist with their physiological response and together condition disease severity. Among these, canopy architecture and phenology are especially influential because they shape exposure, within-canopy microclimate, recovery capacity and the timing of host–pathogen interaction. This has important implications for both genetic inference and selection, and these two groups of traits are therefore considered separately below.

#### 3.4.1. Architectural Traits as Sources of Bias

Numerous studies have shown that Ascochyta blight severity and architectural traits do not segregate independently in biparental populations or breeding programs [65,84,96,97,98,99,100,101]. This association is likely due to several mechanisms that can modulate the observed disease severity. Canopy architecture can act at two different levels: by shaping the plant microenvironment and by conditioning secondary inoculum dispersal. Closed canopies can favor disease development as they ensure optimal humidity conditions for colony growth and fructification. In addition, dense canopies show increased proximity between healthy and infected organs within the plant. However, this can also pose a physical barrier to conidial dispersal to neighboring plants [12,102].

Basal branching can also influence Ascochyta blight severity through two different routes. On the one hand, it modulates canopy density (and, indirectly, its associated effects already discussed), but it also determines recovery capacity after Ascochyta blight damage. In seasons when epidemic progression does not continuously keep pace with plant growth, genotypes with greater branching can generate new healthy biomass after early damage. Thus, the apparent disease severity at the canopy level can be reduced. Consistently, Castro-Urrea et al. [98] reported an additive genetic association between Ascochyta blight severity and the number of basal branches, using multivariate mixed models.

Greater height has also been associated with lower disease severity [65]. One possible explanation is an apparent recovery effect similar to the one proposed for branching, although in this case, the production of healthy biomass occurs through apical growth. However, in controlled assays, this relationship between height and disease severity has been reported to be reversed [103].

Lodging is the tendency of the plant to lean on the ground surface instead of maintaining an erect growth habit. This trait has long been considered a key confounder in field Ascochyta blight assessments. Lodging increases canopy contact with the soil, extending wetness duration and favoring disease development and spread. It also compresses lower canopy layers that experience poorer ventilation [104]. As a result, observed severity can covary both with standing ability itself and with traits that contribute to the erect/lodged phenotype (e.g., stem thickness and lignification level, plant height, internode length, leaf type) [84]. In particular, the semi-leafless (*afila*) pea types are often associated with better standing ability and more ventilated canopies, both relevant traits that can limit Ascochyta blight progression [105].

Altogether, these confounder traits become critical when they bias the phenotypes intended for genetic mapping of disease resistance trait. Jha et al. [65] argue that loci and genetic markers that have been associated with resistance in field trials may partly reflect variation in lodging and height. This aligns with reports of colocalization between genetic regions controlling standing ability, height, and branching and those controlling Ascochyta blight severity [99,101]. Such overlaps may reflect true pleiotropy or tight linkage between distinct loci, but it is also plausible that they are driven by architectural confounding in the phenotype.

#### 3.4.2. Phenology as a Source of Bias

Phenology can covary with Ascochyta blight severity or incidence in the field. The crop development pattern can shift the synchrony between host development and the climatic window most conducive to the epidemic. Interpreting disease scores requires considering this framework. Several studies have reported covariation between Ascochyta blight pressure and phenological variables, or related management practices [41,100,106,107,108].

Similar to other diseases or pests, organ age modulates the response to Ascochyta blight infection and the dynamics of lesion development. In controlled assays, Richard et al. [11] observed a tendency towards greater susceptibility as tissues matured, particularly with the onset of senescence. Hwang et al. [108] reported consistent observations under glasshouse conditions for whole plants and for foliar tissue. In experiments where infection onset was controlled, the timing of inoculation had an effect on yield components and seed infection ratio [106].

At the management scale, earlier sowing can reduce initial exposure to ascospores released from crop residues. This timing also determines how developed the crop canopy is when weather becomes most conducive to disease, its recovery capacity at that time, and how long the crop remains exposed to those conditions [7,109]. For the same reasons, the timing of epidemic onset is also important. Delaying the artificial inoculation of pea plots by one or two weeks has been reported to reduce overall severity in field trials [108].

Taken together, these phenological effects support the notion of temporal escape as a management tool. As discussed for architectural confounders, these effects pose a risk of bias if disease response is conditioned by phenology or scheduling of the crop. Indeed, several studies have reported colocalization between loci controlling photoperiod response, maturation, and earliness and those associated with Ascochyta blight resistance [96,100,110,111].

## 4. Physiological Basis of Pea Resistance to Ascochyta Blight

**Figure 2:**
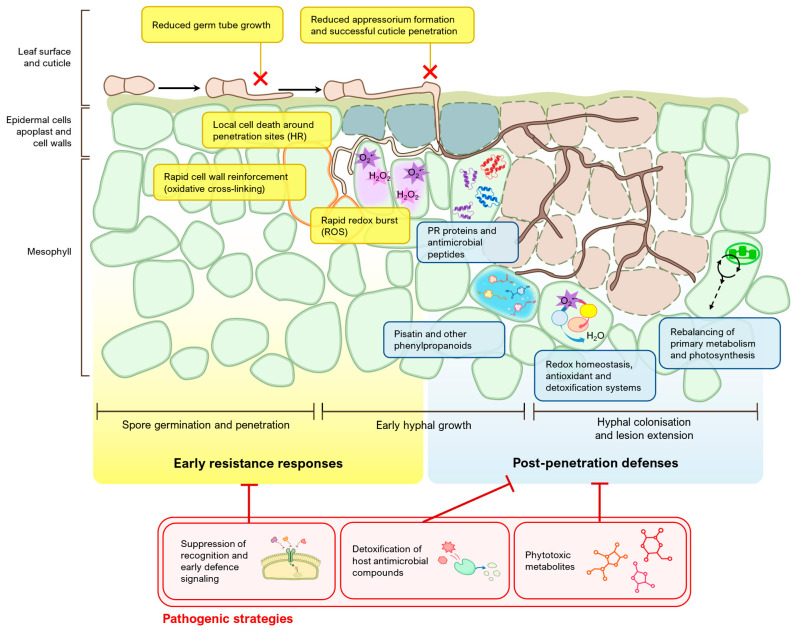
Schematic representation of host resistance responses and pathogen strategies during Ascochyta blight infection in pea. A cross-section of a pea leaf is used as a representative example to illustrate the spatial and temporal organisation of defense responses during infection by Ascochyta blight pathogens. Yellow elements denote early resistance responses, whereas blue elements denote post-penetration defenses. Green plant cells represent living cells, dark green cells represent cells undergoing the hypersensitive response (HR), and brown cells represent cells killed by pathogen activity. ROS, reactive oxygen species (e.g., O_2_.^−^ and H_2_O_2_); PR, pathogenesis-related. Red arrow—inhibition.

Most of the evidence on Ascochyta blight resistance mechanisms in pea comes from studies on *D. pinodes*. In general, the responses described correspond to incomplete, multi-component resistance. Resistance can be decomposed into pre-penetration and early responses (linked to reduced penetration/establishment efficiency or restriction of early mycelial growth), and post-penetration responses (aimed at restricting tissue colonization) [112,113]. Figure 2 provides a schematic overview of these resistance responses and the main pathogen strategies discussed below.

### 4.1. Early Resistance Responses

In leaves, a certain level of resistance to *D. pinodes* can be expressed from the penetration phase, reducing the number of successful infection points. Early studies described differences in the frequency of appressorium formation and penetration success between partially resistant and susceptible pea genotypes. These events at the epidermal interface are modulated by the experimental context, particularly the organ/tissue infected, inoculum density, and the microclimate at the leaf surface [112].

Reduction in penetration success and early limitation of hyphal development after entry have been observed in other legume species infected by *D. pinodes* [114]. In pea, partial resistance to *A. koolunga* has been associated with shorter germ tubes and lower establishment success of conidia, both in leaves and stems [115].

Partially resistant accessions can also show local responses in epidermal cells around penetration sites, including cell death, redox reactions and rapid cell wall reinforcement (e.g., oxidative cross-linking of proteins). These responses are especially relevant during the early establishment phase, when infection hyphae may remain associated with the epidermal wall or subcuticular region before extensive necrotrophic colonization develops. Altogether, these mechanisms contribute to restricting penetration and colony establishment [113].

### 4.2. Multi-Component Post-Penetration Defense

At infection sites where penetration and early establishment are not fully prevented, the plant can deploy a set of parallel and complementary defense mechanisms. Most of these mechanisms demonstrate modest, though potentially cumulative, effects that tend to be sustained over time.

Among them, pisatin and other phenylpropanoid compounds play a major role as phytochemical defenses [116]. Notably, their effectiveness depends on host physiological status and on the pathogen’s capacity to tolerate them [117].

Redox processes also contribute to post-penetration resistance. They involve the control of reactive oxygen species (ROS) and the maintenance of redox homeostasis through antioxidant and detoxification systems. This balance may preserve defense redox signaling while limiting excessive oxidative damage and cell death [118].

A further group of defense mechanisms in pea response to Ascochyta blight involves pathogenesis-related (PR) proteins and antimicrobial peptides. Chitinase accumulates in pea leaves after infection, while the expression of defensin antimicrobial peptides is increased [119,120].

Beyond defense and stress-associated responses, the rebalancing of physiological processes such as primary metabolism and photosynthesis is relevant in Ascochyta blight resistance [121,122,123].

### 4.3. Pathogenic Strategies of Ascochyta Blight Fungi

Pathogen virulence factors interfere with early host defenses as suppressors that attenuate elicitor-induced responses. Such suppression mainly targets oxidative and defense processes in the apoplast and the membrane [124]. The Ascochyta blight pathogens are known to detoxify host antimicrobial metabolites, which may undermine the effectiveness of chemical defenses [117]. Under a necrotrophic strategy, Ascochyta blight pathogens produce phytotoxic secondary metabolites, notably pinolidoxin, 7-epi-pinolidoxin, 5,6-dihydropinolidoxin, 5,6-epoxypinolidoxin, pinolide, herbarumin II, 2-epi-herbarumin II, ascochitine, and ascosalitoxin. These compounds promote necrosis and physiological disruption, contributing to symptom development [125,126,127,128,129,130].

### 4.4. Potential Breeding Value of Physiological Resistance

From a breeding perspective, it is useful to weigh these mechanisms by their field stability across changes in inoculum composition across seasons and locations [8,9]. In addition, an effective and useful defense against Ascochyta blight must involve a localized and temporally appropriate activation, followed by control of self-damage.

These considerations lead to a set of priorities for the design of founding crosses for breeding programs. First, resistance mechanisms that act rapidly and locally at infection sites are valuable as a first defense layer, hampering tissue disruption and/or colony establishment (i.e., apoplast and cell wall modifications) [112,113,131,132]. As a second layer, resistance mechanisms that reduce the rate of lesion expansion can be strong complements (i.e., antimicrobial metabolism, detoxification, and redox control) [96,121,133]. Pyramided together, these elements would lead to a partial resistance phenotype capable of sustaining functionality under infection pressure.

## 5. Genetic Architecture of Pea Resistance to Ascochyta Blight

### 5.1. Genetic Evidence for Resistance to Didymella pinodes

Early genetic analyses showed that the pea response to *D. pinodes* is organ-specific. Under controlled inoculations of F2 populations, disease responses in leaves and stems segregated independently, suggesting differentiated genetic control [53]. This fact reinforced the practical need to evaluate resistance by organ and symptom type, instead of treating it as a single trait [112]. The quantitative inheritance of resistance to *D. pinodes* was demonstrated through diallel and triple test cross analyses that detected additive and dominance effects in the genetic architecture of the trait [134]. Early mapping confirmed an oligogenic/polygenic inheritance, with multiple loci of small to moderate effect [51,110].

Two F2-derived populations were developed from crosses between the partially resistant breeding lines A26 and A88 with the susceptible cultivar Rovar. In these populations, numerous quantitative trait loci (QTLs) with modest effects were detected [110]. These QTLs were mapped across the seven linkage groups (LGs I–VII) using random amplified polymorphic DNA (RAPD) [135], restriction fragment length polymorphism (RFLP) [136], amplified fragment length polymorphism (AFLP) [137], and sequence-tagged site (STS) [138] markers. Some resistance QTLs were detected repeatedly (e.g., Asc2.1, Asc3.1, Asc5.1, Asc7.1), whereas others were trait- or population-specific [110].

Later studies evaluated a recombinant inbred line (RIL) population derived from the cross Carneval (partially resistant to *D. pinodes*) × MP1401 (susceptible) across multiple environments. They were able to map several QTLs on LG II, IV and VI using AFLP, RAPD and STS markers. QTLs associated with resistance together explained ~36% of the variance in the across-environment response to *D. pinodes* [99]. In a different RIL, derived from DP (partially resistant to *D. pinodes*) × JI 296 (susceptible), six QTLs were associated with seedling resistance under controlled conditions (~74% variance explained) and ten QTLs for adult plants in the field (~57–67% depending on trait/organ). Some loci were shared between developmental stages [111]. These QTLs were later repositioned across several linkage groups using higher-density maps, namely LG II, LG III, LG V (Va), LG VI and LG VII [65].

In a RIL population derived from the interspecific cross P665 (*P. sativum* ssp. *syriacum*, partially resistant) × Messire (*P. sativum*, susceptible), Fondevilla et al. [139] identified QTLs associated with resistance to *D. pinodes*. The associated loci were mapped on a linkage map based on simple sequence repeat (SSR) markers [140]. The associated marker MpIII.1 (LG III) was of particular interest, as it was detectable under both controlled and field conditions, and explained ~29% of phenotypic variance. A later analysis implementing a denser marker set and a multi-trait approach identified additional resistance QTLs on LG III and LG VI, within the same RIL population. Some of them colocalized with QTLs for phenological and architectural traits [141]. Later, the combination of a linkage map with high density of single-nucleotide polymorphisms (SNPs) [142] and histological phenotyping further resolved the genetic basis of cellular resistance responses and identified additional associated regions, including MpII.1 (LG II), MpIII.5 (LG III) and MpV.2/MpV.3 (LG V) [143].

In a different interspecific RIL derived from the cross Alfetta (*P. sativum*, susceptible) × P651 (*P. fulvum*, partially resistant), high-density SNP mapping positioned QTLs on several linkage groups (LG I-IV, LG III and LG VII). These loci showed low-to-moderate phenotypic effects that depended on environment and plant developmental stage [100]. In this genetic background, abIII-1 (LG III) was the most consistently detected across stages and environments, and explained ~28% of the variance. In a further study, a heterogeneous inbred family (HIF) population was developed from the same cross. With the use of fine mapping, two independent association signals were resolved within the interval abI-IV-2 (i.e., abI-IV-2.1 and abI-IV-2.2), explaining ~5.5–14% and ~7–10% of variance, respectively [65].

A genome-wide association study (GWAS) based on SNP markers reported significant marker–trait associations (MTAs) for resistance to *D. pinodes* under controlled conditions, using phenotypic data of disease progression. These MTAs mapped to several chromosomes, including Chr1/LG VI (~26 and ~369.9 Mbp), Chr5/LG III (~198 Mbp), and Chr7/LG VII (~37.5 and ~336.9 Mbp) [103]. An independent GWAS using Diversity Array Technology (DArT) markers reported a major locus in Chr2/LG I for seedling resistance within an interval of ~8 Mbp containing multiple annotated genes, including serine/threonine kinase-type candidates [57].

Taken together, these mapping studies indicate that consistency tends to appear at the level of broad genomic regions. While LG III and LG VII are repeatedly pointed out across populations, the variation between phenotyping settings and marker frameworks limits fine testing of colocalization. Likewise, interspecific sources can contribute additional signals that are not readily aligned to earlier *P. sativum* backgrounds. Comparative alignment using shared landmarks (including candidate genes and physically mappable markers) becomes necessary to consolidate the genetic landscape [65,100,101,144].

The multi-population synthesis provided by Boutet et al. [101] reduced fragmentation in the genetic evidence. The authors defined five recurrent meta disease architecture frost (MDAF) regions that captured an important fraction of the QTLs reported in the earlier literature (i.e., MDAF.3.1, MDAF.3.2, MDAF.5.1, MDAF.5.2, and MDAF.6.2). In particular, this study demonstrated that the resistance-associated region repeatedly detected on LG III across studies does not represent a single peak. Instead, this methodology decomposed it into at least two distinct genomic windows. Similarly, on LG V and LG VI, previously reported resistance QTLs converged within narrow consensus regions. On LG VII, the evidence pointed to at least two subregions defined by specific sets of mappable markers and/or resistance gene analogues (RGAs).

To integrate mapping and GWAS results within a common reference system, the chromosome-scale assembly of Kreplak et al. [145] is the most convenient platform. This is the reference assembly used by recent studies reporting physical positions and candidate regions associated with resistance to *D. pinodes* [57,101,103]. These GWAS studies identified consistent MTAs beyond the consensus regions previously discussed. Some association signals fall outside MDAF windows on LG III/V/VI, which may reflect additional genetic variation in the broader GWAS panels and/or dependence on phenotyping design.

### 5.2. Genetic Evidence for Resistance to Other Ascochyta Blight Pathogens

Compared with the volume of genetic evidence for resistance to *D. pinodes*, the case of other complex members is much more limited. The available landscape consists of isolated inheritance studies and a small number of association signals [14].

For *D. pinodella*, it has been suggested that resistance to foliar disease and foot rot syndrome is independently inherited. Across a broad panel of pea genotypes infected with *D. pinodella*, no significant correlation was found for these traits [35]. The clearest insights into the genetic architecture of resistance to this pathogen come from classical inheritance work. In crosses between the resistant cultivar Kinnauri and several susceptible lines, segregation for resistance suggested a major dominant gene governing the inheritance of the trait [146]. More recent studies confirmed the genetic independence of resistance to *D. pinodella* and resistance to *D. pinodes* [58].

In *A. pisi*, inheritance studies point to qualitative resistance under controlled inoculation. In the cross Ottawa A-100 (resistant breeding line) × Thomas Laxton (susceptible cultivar), the segregation pattern for resistance to *A. pisi* was attributed to two dominant genes, each capable of conferring resistance on its own [69]. In an independent F2–F3 population genotyped with RFLP/RAPD markers, associated regions were mapped. Most of the variation was explained by three genetic intervals and a major locus on chromosome 4 [147]. However, the genetic evidence base remains limited for this pathogen and has not matured into modern, widely validated markers.

For the response to *A. koolunga*, genetic evidence is restricted by its recent recognition as a pea pathogen. However, a GWAS study has recently identified a locus on Chr5/LG III associated with seedling resistance, defining a genetic window of 35 Mbp with several candidate genes annotated (e.g., protein kinase-encoding genes) [57]. The physical map position of this MTA does not correspond to any of the consensus regions delimited by Boutet et al. [101] for resistance to *D. pinodes*.

The list of loci associated to date with resistance to Ascochyta blight in peas is synthesized in Table 3, which locates the genetic markers within a common reference framework of consensus genomic regions.

### 5.3. Consistency and Stability of Resistance Loci

Taken together, the genetic evidence discussed here indicates that resistance to Ascochyta blight is mainly quantitative, and current knowledge remains centered on *D. pinodes*. The consistency of these genotype–phenotype associations depends on whether their effects persist across experimental contexts, environments, and genetic backgrounds. Early comparative analyses already showed that the detected effects depended on the phenotypic variable and organ assessed, with limited consistency between populations [110].

Some experimental designs address stability across environments (e.g., growing seasons and locations) through multi-environment trials or through parallel evaluations in the field and under controlled conditions. These analyses often separate a small number of stable loci from a larger set of loci that are environment-dependent. In general, recurrence of genetic associations is usually observed at the level of broad regions [99,100,111]. In the field, the genetic associations can be partly conditioned by confounding traits or covariates that modulate exposure and epidemic progression. This fact can lead to colocalization of resistance and agronomic traits, as reported in some studies [99,101,141].

Another point to address is validation across genetic backgrounds, namely, whether a locus retains an effect in a different genetic context and at different allele frequencies. Recurrence across genetic backgrounds is often limited to broad regions or linkage groups. Genetic associations reported by different studies or mapping populations may localize to different positions, consistent with allelic heterogeneity and background dependence [65,100]. Non-additive interactions (epistasis) between QTLs for resistance to *D. pinodes* have also been proposed [148]. When assessing transferability across genetic backgrounds, validation schemes should consider haplotypes and multi-locus combinations when the phenotypic effects of some loci depend on epistatic combinations.

In practice, stability should be evaluated at the level of comparable regions. Validation across environments is particularly important. Also, comparing the behavior of these associations across different developmental stages and genetic backgrounds remains strongly informative. Finally, contrasting controlled and field settings provides a valuable approach to distinguish consistent associations.

## 6. The Pea–Ascochyta Blight Pathosystem at the Molecular Scale: Biochemical Insights and Omics Approaches

### 6.1. Targeted Biochemical Insights

A substantial part of the knowledge about the pea–Ascochyta blight pathosystem was built from targeted biochemical and enzymatic assays. Taken together, these studies point to fungal signals that can activate defense responses and, in parallel, a set of pathogenicity factors that delay or disrupt them (as already introduced in Section 4).

A well-characterized case is the elicitor/suppressor system of *D. pinodes*. A pattern of lipid phosphorylation involving polyphosphoinositides has been documented in plasma membrane fractions in response to a fungal elicitor [149]. This early interference is consistent with the differential regulation of mitogen-activated protein (MAP) kinases reported in response to *D. pinodes* signals. In this regulation, the elicitor and suppressor show contrasting effects on kinase activation [150].

Alongside these signaling events, the cell wall and the apoplast constitute a functional module in which redox signaling is generated and regulated. In solubilized cell wall extracts, superoxide anion generation was observed and attributed to cell wall peroxidases. In multi-host comparative assays, this system responded to *D. pinodes* elicitor stimuli in a host-non-specific manner. By contrast, suppression of the redox response by the pathogen-derived suppressor occurred only in pea. This suggests that part of the compatibility of the interaction may be established during the earliest stages of infection [131]. In the same pathosystem, cell wall ATPase complexes were reported to couple to hydrogen peroxide production via a copper amine oxidase. This coupling was also sensitive to elicitor/suppressor signals from *D. pinodes*, supporting the idea of an integrated apoplastic hub linking recognition with redox dynamics [132]. The identification of PsAPY1 (NTPase) was proposed as a potential component of microbial signal recognition. This is consistent with a model in which extracellular receptors and enzymes couple pathogen perception to ROS generation and regulation [151].

Furthermore, fungal suppressors from *D. pinodes* have been shown to inhibit the pea plasma membrane H^+^-ATPase activity [152]. Consistently, Amano et al. [153] showed that, in vitro, the fungal suppressor markedly inhibited both ATPase activity and proton transport. Together, these findings support the idea that pathogen-mediated disruption of ion and pH homeostasis contributes to local susceptibility [152,153]. These findings have been reviewed and synthesized into a model of fungal suppressors that clarifies how elicitor/suppressor factors interfere with basal functions in pea [124].

Studies focused on PR-type responses provided interesting insights into the pathosystem. Vad et al. [119] showed that chitinase activity increased in pea leaves inoculated with both virulent and avirulent isolates of *A. pisi*. They purified three chitinase isoenzymes, which later became widely used markers of induced antimicrobial activation in pea. In addition, recent targeted assays highlighted redox metabolism and defense components as tightly associated with incomplete resistance in interactions of pea with *D. pinodes*, *D. pinodella* and *A. koolunga* [58,154].

Another well-established line of biochemical research focused on the chemical interplay between the plant-produced defense compounds and fungal phytotoxic metabolites. Pisatin was isolated and characterized as a key pea phytoalexin [155]. Subsequent studies showed early accumulation of pisatin after inoculation with *A. pisi*, with higher concentrations in the brown tissue adjacent to lesions [156]. Comparisons between lesions with restricted and active expansion suggested that physiological and microenvironmental conditions can influence the accumulation and effectiveness of the compound [116].

On the pathogen side, Yamada et al. [157] showed that the combined presence of *D. pinodes* elicitor and suppressor agents delayed the accumulation of certain transcripts. This was the case of the transcripts encoding phenylalanine ammonia-lyase and chalcone synthase, two enzymes that catalyze early steps in the biosynthetic pathways of phenylpropanoids and flavonoids/isoflavonoids, respectively. Within the pathogenic strategy, an effective suppression of host response would extend the time window available for colony establishment [157]. Pisatin degradation has been documented in *D. pinodes* and, to a lesser extent, in *A. pisi*. This capacity was shown to depend on culture conditions, including the carbon source, suggesting regulatory control of the degradative machinery [158,159]. At the enzymatic level, pisatin degradation was linked to cytochrome P450 induction in various pathogens within the complex [117]. Consistently, although *A. pisi* is sensitive to pisatin, this compound did not slow lesion expansion of *D. pinodes* under a humid microenvironment [7].

Like other fungal pathogens, Ascochyta blight fungi produce a range of phytotoxic metabolites that could act as phytotoxic compounds, contributing to the development of disease symptoms in plants [125,126]. These metabolites belong to different classes, including macrolides, polyketides, anthraquinones, meroterpenoids, solanapyrones and peptides. Phytotoxins described from fungal isolates cultured on different growing media include pinolidoxin [128], pinolide, herbarumin II, and 2-epi-herbarumin II [127,129,130]. While pinolidoxin exhibited strong phytotoxicity across multiple plant species, the other nonenolides showed no significant effects, highlighting the importance of stereochemistry in their activity [130]. Evidence for the production of these metabolites *in planta* remains limited, constraining interpretation of their ecological roles and their contribution to disease development under field conditions.

### 6.2. Genomic Resources

In recent years, efforts to map resistance-associated loci have evolved towards genomics-supported approaches, as anticipated by Rubiales and Fondevilla [160] for Ascochyta blight in legumes and in line with broader breeding trends [93,161]. This change has been enabled by two parallel developments: the consolidation of host genomic resources, together with the recent emergence of genomic resources for members of the pathogenic complex.

Before a pea reference genome was available, genotyping resources and platforms had already been developed to a reasonable degree. Pea-specific SNP arrays and saturated consensus maps enabled mapping in diversity panels and biparental populations [162,163,164]. This progress was further supported by the first reference genome for *P. sativum*, which enabled linkage groups and QTLs to be anchored to physical chromosomal positions [145]. This was followed by improved genomic representations that captured structural variation and gene-content diversity in germplasm, from a pan-genomic perspective [165].

In practice, these resources reduced ambiguity when comparing QTLs across studies and facilitated the identification of functional candidates from the pool of predicted genes within the associated regions. Building on this, GWAS studies localized loci associated with seedling resistance to *D. pinodes* and *A. koolunga* on defined chromosomal regions. Functional candidates annotated within those intervals included genes encoding signaling elements, like serine/threonine kinases and ethylene-responsive kinase homologs [57,103].

On the pathogen side, long-read assemblies and comparative pan-genomic analysis have clarified patterns of genetic diversity across the pea Ascochyta blight complex, with genome assemblies available for *D. pinodes*, *D. pinodella* and *A. koolunga* [166]. Furthermore, other efforts have led to the construction of a near-chromosome-level assembly of the *D. pinodella* genome [167] and a high-quality annotated genome of *A. pisi* [168]. In addition to these insights in taxonomic resolution, these comparative assemblies also provide a starting point to profile candidate virulence factors, including secreted proteins, carbohydrate-active enzymes (CAZymes) and secondary metabolite biosynthesis gene clusters. These resources also help to distinguish conserved and accessory components within the pathogenic repertoires [166,168].

These pathogen genomic resources enable more comparable analyses of pathogen virulence and host defense mechanisms, and improve the interpretability of transcriptomic, proteomic, and metabolomic analyses in the pea−Ascochyta blight pathosystem. From a breeding perspective, structural and gene-content variation within Ascochyta blight pathogens warrants close attention. It may lead to variation in virulence strategies, with potential consequences for the durability of host resistance [166,169].

### 6.3. Transcriptomics

Transcriptomics was the first omics approach to be used in the pea−Ascochyta blight pathosystem, which led to a better characterization of host transcriptional reprogramming under infection. Most transcriptomic evidence in pea Ascochyta blight derives from *D. pinodes* infection and is based on experimental designs comparing the resistant accession P665 and the susceptible Messire at early stages of infection [96,133].

An initial study based on a heterologous microarray using *Medicago truncatula* oligonucleotide probes identified 346 transcripts that were differentially expressed in infected P665 relative to Messire. These differentially expressed genes (DEGs) were assigned to multiple functional categories, including a relevant subset associated with defense/cell rescue processes and primary metabolism [96]. Subsequently, deepSuperSAGE profiling identified 17,561 UniTags, 509 of which were differentially expressed in the same genotype comparison [133]. Across these sets, consistent functional patterns included transcripts associated with cell wall modification (e.g., cell wall reinforcement and lignification), redox processes (e.g., oxidative protein cross-linking), and the synthesis of apoplastic defense compounds. The observed changes also involved signaling-associated transcripts, suggesting hormonal rebalancing during infection. These included regulation of jasmonate/ethylene signaling, the involvement of abscisic acid (ABA) and auxin pathways, and repression of the gibberellin pathway. Defense-associated candidates discussed in these studies also included a PR14 lipid-transfer protein, the defensin precursor DRR230-b, and antimicrobial response genes [96]. As a complementary source of evidence, targeted analyses of pea defensin genes have also indicated pathogen-induced expression in response to *D. pinodes*, including members of the DRR230 family [120].

The cited studies faced a shared limitation in their reliance on a single resistance source and a specific experimental design. In transcriptomics, a substantial fraction of DEGs can reflect general responses to physiological stress and tissue damage. In addition, some patterns may be inherent to the assessed tissue, sampling time, or host developmental stage. These factors limit the extrapolation from an isolated resistant/susceptible comparison to other genetic backgrounds or conditions [170]. To address these limitations, one practical strategy is to integrate them with mapping insights, and focus on candidates supported by both approaches. On this logic, Fondevilla et al. [171] selected ten candidate genes previously shown to be induced by *D. pinodes* infection in P665 and mapped them in the P665 × Messire RIL population. Five showed significant associations with resistance traits and colocalized with genomic regions previously associated with these traits. In the same RIL population, a further transcriptomics approach combined bulk-segregant analyses and RNA-seq (bulk segregant RNA sequencing, BSR-Seq). This scheme, which was based on resistant and susceptible bulks assembled from extreme F2 individuals, led to the identification of DEGs whose expression was more consistently associated with resistance [67]. Population-based strategies like BSR-Seq can help to distinguish the genuine resistance-associated transcripts from background noise typically found in simple pairwise comparisons.

Notably, transcriptomic evidence remains limited for other members of the Ascochyta blight complex. In *D. pinodella*, a recent study combined genomic resources with temporal transcriptomics to explore the infection process in resistant and susceptible cultivars. Similar gene repertoires were mobilized by the pathogen in compatible and incompatible reactions, although they differed in timing and intensity. The resistant pea genotype showed stronger induction of defense-related genes [167]. For *A. koolunga*, transcriptomic evidence of host response comes from targeted analyses of selected transcripts. Tran et al. [154] compared infected stems and leaves from resistant and susceptible genotypes and reported contrasting responses between the two organs. In this study, the defensin precursor DRR230 was downregulated in both genotype classes following inoculation with *A. koolunga*, but not with *D. pinodes*. Transcripts associated with pisatin biosynthesis were also more strongly expressed in leaves of the resistant genotype inoculated with *A. koolunga* [154].

### 6.4. Proteomics

Proteomics provides complementary evidence in the pea–Ascochyta blight pathosystem by capturing levels of regulation that are not directly reflected by transcriptomics, including post-translational modifications and protein turnover. This is particularly relevant in necrotrophic interactions, where rapid oxidative stress, cell death, and tissue disruption can distort the correspondence between mRNA abundance, protein accumulation, and functional activity [172].

The first proteomic studies in this pathosystem examined differential protein accumulation in pea tissues infected by *D. pinodes* using two-dimensional electrophoresis (2-DE) workflows. They identified changes associated with infection in the accumulation of proteins linked to defense and stress responses, including PR10, the ABA-responsive protein ABR17, annexin-like, and heat-shock proteins. They also detected shifts in enzymes of central metabolism such as fructose-bisphosphate aldolase, malate dehydrogenase and glutamine synthetase. Proteins associated with the photosynthetic machinery were also altered, including OEE1/OEE2, light-harvesting proteins and RuBisCO [121]. These patterns suggest that incomplete resistance involves not only defensive responses but also metabolic readjustment and maintenance of cellular function under infection pressure.

Subsequently, a quantitative approach based on liquid chromatography–tandem mass spectrometry (LC-MS/MS) was used to profile constitutive protein abundance in segregant individuals from the Messire × P665 RIL population, with the aim of identifying candidates whose abundance levels covaried with resistance [173]. The resulting peptide panel included proteins associated with cell wall modification, calcium signaling, redox and sulfur metabolism, proteostasis, translation, and mitochondrial function. These findings are relevant as they help to define proteomic patterns associated with basal resistance, enabling the identification of candidate proteins and processes that may contribute to sustained functionality during infection.

Interpretation of proteomic evidence is nevertheless sensitive to experimental design and methodology. Proteomic coverage depends on extraction and solubilization protocols, which can bias the classes of proteins that are recovered, and gel-based approaches like 2-DE are particularly limiting for membrane proteins [172]. In addition, proteomic profiles depend on the tissue and developmental stage of the plant, so that apparently constitutive differences may partly reflect underlying physiological states of the host, mixed with the resistance features [174].

Another line of proteomic studies in pea Ascochyta blight examined infection in the broader physiological context of beneficial microsymbioses. In leaf multi-omics studies, the presence of symbionts was associated with consistent changes in protein synthesis metabolism, metal homeostasis and redox processes. Rhizobial symbiosis was reported to elicit induced systemic resistance to *D. pinodes*, with higher levels of proteins involved in pisatin biosynthesis [175]. Similar studies that focused on seeds identified additional functional candidates for disease resistance. In plants inoculated with *Rhizobium* and subsequently challenged with *D. pinodes*, proteins that contributed most to the differences between treatments included late embryogenesis abundant (LEA) proteins, specifically, dehydrins, and enzymes associated with carbohydrate metabolism and defense. In turn, storage proteins such as vicilins showed genotype-dependent behavior [122]. An integrative study in plants colonized by arbuscular mycorrhiza corroborated the relevance of LEA proteins during *D. pinodes* infection. It also pointed to changes in storage proteins, a lower representation of plastid-related proteins, and an increased representation of proteins linked to signaling/stress, glycolysis, and flavonoid/isoflavonoid metabolism in the more tolerant genotype [123]. Altogether, these findings contribute to broadening the molecular knowledge of the pea–Ascochyta blight pathosystem. However, their multifactorial experimental designs complicate direct comparisons across studies and the extrapolation of candidate proteins as operational markers.

### 6.5. Metabolomics

Metabolomic approaches offer an informative link between plant genotype, physiology, and phenotype. However, the interpretation of these studies requires careful consideration of experimental factors such as tissue assessed, developmental stage of the plant and environmental conditions. All of these factors potentially modulate the observed chemical profile [176,177].

A substantial part of the metabolomic evidence for resistance to Ascochyta blight comes from the multi-omic studies discussed above. Desalegn et al. [175] and Turetschek et al. [118] analyzed *D. pinodes* infection in leaves under different microsymbiotic treatments. Among all the experimental conditions, the lowest infection rates and slowest disease progression were observed in plants treated with rhizobia prior to inoculation with *D. pinodes*. In those plants, the citric acid cycle, the pisatin pathway, amino acids, and secondary metabolism were markedly regulated [175]. However, although symbionts modified the response to infection, the host genotype proved a stronger influence on disease tolerance. At the metabolic level, tolerant genotypes exhibited a maintenance of photosynthetic function, a continued supply of metabolic precursors for defense metabolism, and redox homeostasis sustained through sulfur metabolism and the glutathione-ascorbate system. Modulation of jasmonate/ethylene processes may also help to limit the induced cell death in these genotypes [118].

Studies of the seed metabolome extended this framework to the systemic consequences of infection and symbiosis. According to these studies, rhizobial inoculation altered the metabolome of mature seeds produced by treated plants. The observed changes affected glycerophospholipid compounds and specialized metabolites. Notably, the stress-related oligoglycoside pisumoside B increased in plants with rhizobial symbionts. Meanwhile, under *D. pinodes* infection, the specialized secondary metabolites Soyasapogenol C, Api_Dai_Kae_Flavon and a 6-Hydroxyapigenin derivative showed stronger accumulation in a tolerant pea genotype [122]. A subsequent study with broader metabolite coverage also examined arbuscular mycorrhizal symbiosis. It revealed changes across treatments in several classes of seed metabolites, including flavonoids/isoflavonoids, anthocyanin-like pigments, prenol lipids and derivatives of carboxylic acid. The results also reflected changes in metabolites associated with defense and stress signaling. This was the case for ABA, which increased in infected seeds relative to healthy seeds, irrespective of symbiotic treatment. In turn, N-jasmonoylisoleucine concentrations decreased in all the infected seeds [123]. Taken together, these findings suggest that seed responses to infection and symbiosis are strongly genotype dependent.

A recent study explored the metabolomic aspects of novel fungicide seed treatments against pea Ascochyta blight. Stałanowska et al. [178] investigated the effect of seed coating with silver nanoparticles on the subsequent response of the seedling to *D. pinodes*. Seed treatment reduced disease severity and pathogen load. The seedling protection showed a distinctive metabolic signature, which included changes in primary metabolism, particularly in amino-acid and carbohydrate profiles.

On the pathogen side, recent advancements in analytical platforms, particularly untargeted and targeted metabolomics, have shown a possible effector-like role for pinolidoxin in promoting early infection through interference with host signaling pathways [179]. Untargeted metabolomics of the host revealed cultivar-specific metabolic responses emphasizing the complexity of host–pathogen interactions and the importance of multi-omics approaches for identifying molecular targets for deeper systems-level insight into pea defense strategies.

Untargeted metabolomics can also be applied to chemotaxonomy. As an illustrative example, liquid chromatography-mass spectrometry (LC-MS) was used to profile metabolites of culture extracts from several Ascochyta blight pathogens of legumes. This study demonstrated high within-species similarity alongside marked inter-species divergence in metabolic features. Hierarchical clustering resolved five major chemical groups that were largely consistent with genetic phylogeny. However, closely related taxa such as *D. pinodes* and *D. pinodella* were chemically indistinguishable in this study. Interestingly, Ascochitine was a distinctive metabolite of the chemical group centered on *A. pisi.* It was also detected in strains of *A. koolunga*. Other known metabolites likewise showed the expected taxonomic distribution, including pinolidoxin in *D. pinodes* [180].

## 7. Molecular Breeding for Ascochyta Blight Resistance in Pea

### 7.1. Marker-Assisted Selection

Genotype−phenotype associations (QTLs, MTAs, and omics insights) are a starting point for the development of breeding tools, but their practical value depends on the stability of the underlying associations across different environments, inoculum composition, and epidemic trajectories. In legumes, the uptake of marker-assisted selection (MAS) has been relatively slow, and its use is limited. One reason is that markers suitable for routine use in diverse breeding programs and target environments are difficult to obtain [181]. In pea resistance to Ascochyta blight, a similar pattern is observed. Despite the number of resistance QTLs reported, only a few of them have shown consistent effects across environments. As a consequence, the pool of markers with sufficient support for validation in breeding material remains small [93].

Nevertheless, the reduced set of markers that are supported by consistent associations still requires validation to be considered for breeding use. An illustrative effort of marker validation for Ascochyta blight resistance in cultivated germplasm is provided by Jha et al. [182]. In this work, a small set of SNP markers was genotyped using KASP assays in a panel of 36 pea cultivars from the Saskatchewan regional variety trial. The authors tested the association of marker alleles with Ascochyta blight incidence across a large number of site-year environments. Among the SNP markers analyzed, RGA-G3Ap103 and PsC8780p118 stood out for higher consistency, with significant associations in various site-years, while other markers were either less recurrent or inconsistent across environments [182].

Given the limitations discussed above, MAS is not yet suitable for routine use in breeding for Ascochyta blight resistance in pea. At present, it is more useful for specific tasks. These might include tracking the inheritance of resistance-associated alleles within specific breeding populations and supporting the accumulation of different resistance traits [182,183].

### 7.2. Genomic Selection

As a complementary approach, genomic selection (GS) uses genome-wide marker information to predict the genetic value of breeding lines. In contrast to MAS, which targets a limited number of markers with phenotypic effects, GS captures and models the cumulative effects of a large number of loci simultaneously [184]. Prediction accuracy is strongly influenced by phenotypic data. It is also affected by the degree of relatedness between the training set and the selection candidates, and whether the training set sufficiently captures genotype-by-environment (G×E) interactions present in the training set [185,186].

In pea, only one study has directly applied GS to Ascochyta blight resistance so far. Carpenter et al. [13] fitted several prediction models for Ascochyta blight disease score using genotyping-by-sequencing (GBS) genomic data, under a cross-validation framework. Additionally, they compared alternative marker-quality thresholds and phenotype definitions across trials. The authors noted that predictive ability depended largely on how phenotypic data from different trials were summarized and combined into across-trial means, or adjusted values [13]. These findings place emphasis on the training phenotype: which sources of variation it captures (i.e., environment, epidemic, yield) and how they are modeled, in particular those that can alter the relationship between disease and agronomic performance [6,187]. More broadly, the same factors also condition the applicability of GS across a wider range of genetic materials and environments [186].

Evidence from other crops illustrates that the integration of yield and stress tolerance within large-scale genomic analyses can lead to simultaneous progress in breeding targets. This also applies to traits often considered to involve trade-offs [188]. In pea, GS studies evaluating predictive ability across Italian environments indicated that yield and protein content can be predicted across diverse conditions, using multi-environment evaluation as a framework for assessing stability [189,190].

### 7.3. Definition of Breeding Targets

Breeding for Ascochyta blight resistance in pea should deliver competitive, stable seed yield under real cropping conditions. Accordingly, a reasonable breeding target would be a cultivar that sustains agronomic performance under variable disease pressure, not simply defined by the minimum final disease severity. This would translate into a stable production and good-quality grain, guaranteed by the maintenance of a functional canopy and by a crop phenology compatible with the target environment [8,93]. This perspective places resistance within a wider breeding target defined by agronomic stability.

More broadly, multi-trait frameworks proposed for biotic stresses in pea coincide in framing breeding as the development of high-yielding cultivars with sufficient stress resilience [93]. This must be reflected in pea breeding programs through the definition of competitive ideotypes in which tissue or physiological resistance to Ascochyta blight is regarded as one of several traits contributing to stable performance under disease pressure [8,16].

Field evidence supports this integrated view. Some studies have reported strong simple correlations between disease severity and yield under epidemic pressure [5,65], indicating that higher resistance levels are in fact aligned with higher yield. Nevertheless, under field conditions, this relationship is partly mediated by canopy structure and crop phenology as modulators of epidemic dynamics.

Modest changes in plant architecture can translate into appreciable differences in disease progression [107,191,192]. Plant lodging during the reproductive phase acts as an intermediate trait connecting crop load, harvest efficiency, and epidemic dynamics. Lodging risk increases with the development of seed biomass during pod filling [105,193]. The impact of Ascochyta blight severity on crop performance also depends on the upward disease progression through the canopy and the loss of functional leaf area at each plant node. Yield penalties are more pronounced in progression patterns that lead to a reduction in radiation interception capacity due to defoliation [194]. Moreover, canopy architecture also conditions fungicide effectiveness [104] and technical factors of the crop management such as mechanized harvest [8].

The practical effectiveness of an integrative breeding perspective is supported by results from recurrent field selection schemes. Incremental accumulation of partial resistance already led to simultaneous gains in Ascochyta blight resistance and seed yield under high disease pressure [195,196].

### 7.4. Integrated Disease Management as a Framework for Deploying Genetic Resistance

Genetic resistance to Ascochyta blight is deployed within a management context that can favor, mask or complement its expression. Integrated disease management defines this agronomic context by shaping the inoculum pressure and epidemic timing under which resistant cultivars are grown [8,16]. This is particularly relevant given that inoculum composition, disease pressure, and environmental conditions can change across growing seasons and geographical regions [23,187,197].

Management practices that modulate exposure to primary inoculum and secondary disease spread directly affect the conditions under which resistance is expressed. Sowing schedule determines the timing and intensity of disease pressure faced by the crop and has proved critical for limiting yield losses [6,109,198,199,200]. In South Australia, Bretag et al. [41] observed marked differences between sowing dates. Late sowing reduced severity, although this also led to a yield penalty. Similar patterns have been described for European winter sowings [107]. For breeding, the critical factor is therefore the synchrony between the developmental stage at which resistance traits are expressed and the epidemic window. Canopy structure is also relevant because it shapes the microenvironment in which infection may occur, and consequently, the disease pressure under which resistance traits are expressed. Higher seeding rates generally increase disease pressure, whereas certain intercropping schemes can reduce disease severity and slow disease progress [12,102,191,201,202]. More open and standing canopies may also enhance fungicide effectiveness through improved penetration within the crop [104].

Epidemic risk prediction further supports the alignment of resistance deployment with seasonal disease risk. Illustratively, the ‘Blackspot Manager’ model integrates meteorology and epidemiological parameters to estimate seasonal epidemic risk, providing support for growers’ management decisions [203,204,205]. This logic has been incorporated into Australian regional crop sowing guides, which frame Ascochyta blight in terms of probability and exposure [88,200,206].

Chemical and biological control measures can also complement host resistance, although their value depends on crop accessibility and the pathogen population context. Routine monitoring of fungicide sensitivity provides an operational basis to detect changes in treatment response and adjust fungicide programs [42]. In commercial plots, agronomic gains and economic returns obtained from fungicide strategies are not uniform, reinforcing the role of risk prediction and genetic resistance within the integrated management strategies [48,207]. Biocontrol agents, novel fungicidal compounds, or novel techniques may also provide valuable complementary tools as they are likely to reduce dependence on chemical control [47,178].

### 7.5. Challenges in Molecular Breeding for Ascochyta Blight Resistance

Pea breeding for Ascochyta blight resistance faces a set of interrelated constraints that shape the research insights, such as resistance sources, genetic associations, and omics findings, as well as their implementation in cultivar development [8,93]. Furthermore, some of these limitations reinforce one another.

One major limitation is the composite nature of the field phenotype, shaped by canopy microenvironment and covariation with architecture/phenology as discussed earlier in this review. This complexity hinders extrapolation between phenotyping systems and limits both early selection and the evaluation of advanced material [8,57,107,208]. The problem is further aggravated by the heterogeneity of recorded variables, scales and evaluation times adopted, as well as variation in experimental microclimate and inoculum designs across trials [8,59]. Other sources of structural variation are G×E interactions and organ/growth stage dependence, which can condition the effective expression of resistance among trials and growing seasons [8,108]. All these reasons make it challenging to translate genotype–phenotype associations and knowledge of the pathosystem into practical breeding tools and decisions.

The multi-species nature of the Ascochyta blight complex, together with variation in aggressiveness among isolates, further conditions the experimental results and breeding outcomes, since they depend strongly on the biotic challenge imposed. Without clear characterization and reporting of inoculum composition, comparability across studies and interpretation of resistance levels remains limited [8,57].

Another practical limitation for breeding is the genetic architecture of Ascochyta blight resistance, which is dominated by multiple loci with small to moderate effects. This is reflected in the fragmented, context-dependent QTL landscape reported for this trait [139,209]. Even when loci show statistically significant association with resistance, their contribution to phenotypic variance is frequently limited [57,93]. Given this framework, large, rapid gains in improvement progress are unlikely. Breeding progress, therefore, depends on incremental accumulation of resistance traits while avoiding agronomic penalties [8,57]. In practice, this often relies on transgressive segregation to assemble parental genetic contributions [209].

## 8. Concluding Remarks: Priorities for the Present and Future of Breeding for Ascochyta Blight Resistance

Ascochyta blight remains a major constraint for pea production, and useful resistance in current germplasm is incomplete and context-dependent. Over time, research on this topic has provided insights that point to a mainly quantitative genetic architecture and multi-component resistance. Most evidence, especially for *D. pinodes*, points to loci of small to moderate effect whose expression depends on organ, developmental stage, and testing context. The broader challenge for crop improvement is therefore to translate these context-dependent insights into breeding decisions that deliver stable performance under variable disease pressure [57,93]. This may be supported by consistent genetic markers, careful phenotype assessment, and ideotype definition aligned with the target cropping context.

From a breeding perspective, this complexity appears in the field as a composite phenotype shaped by heritable physiological resistance, but also by canopy microenvironment, crop architecture, phenology, and the epidemic challenge. As a result, apparent resistance levels can vary substantially across trials, and phenotypic data can be distorted by differences in inoculum composition, scoring criteria, and disease development dynamics. This makes the control of bias essential in field evaluation.

A good starting point is to align phenotyping approaches with the selection target. For Ascochyta blight, it is advisable to define the phenotype by organ, developmental stage, and symptom type, and to document critical variables of the biotic challenge and experimental conditions [57]. Trials under controlled conditions are useful for screening resistance sources, and for assessing early segregating material before subsequent field testing. Calibration across trials is critical for this purpose, reducing unreliable extrapolations that could later translate into limited transferability [57,208].

Developing useful selection markers from omics findings first requires translating context-dependent transcriptomic, proteomic, or metabolomic signals into validated DNA-based markers associated with stable genetic variation [175]. In the absence of such evidence, their most immediate contribution to Ascochyta blight resistance remains analytical. Currently, realistic contributions include the identification, characterization and prioritization of the resistance mechanisms that are most useful for breeding. In this context, pathogen genomic resources contribute to a better characterization of the biotic challenge, which can provide more useful information for the design of resistance ideotypes and other control strategies [166].

For MAS to provide practical support, the basic requirement is the availability of genetic markers that are robust to relevant sources of variation and can be extrapolated to other germplasm sets [182]. It is therefore necessary to invest more effort in marker validation. To this end, multi-environment validation trials with common checks provide an essential baseline [8,93]. In contrast, for GS, practical value depends on the prediction accuracy that can be reached. In turn, it depends on the degree of proximity between the training set and the intended application scenario [13].

## Figures and Tables

**Table 1:** Nomenclature and synonymy of the pea Ascochyta blight pathogens.

EEPO Reference Name ^1^	Current Name ^2^	Nomenclatural Synonyms ^3^	Taxonomic Synonyms ^4^
*Ascochyta pisi*	*Ascochyta pisi* Lib.	*Ascochyta pisi* var. *pisi* *Septoria leguminum* var. *pisorum* *Ascospora pisi* *Ascochyta pisi* f. *pisi*	*Ascochyta pisicola* *Depazea pisicola* *Didymella pisi*
*Ascochyta koolunga*	*Ascochyta koolunga* (Davidson, Hartley, Priest, Krysinska-Kaczmarek, Herdina, McKay & Scott) L.W. Hou, L. Cai & Crous	*Phoma koolunga*	–
*Didymella pinodella*	*Didymella pinodella* (L.K. Jones) Qian Chen & L. Cai	*Ascochyta pinodella* *Phoma medicaginis* var. *pinodella* *Phoma pinodella* *Peynorellaea pinodella*	*Phoma trifolii*
*Didymella pinodes*	*Didymella pinodes* (Berk. & A. Bloxam) Petr.	*Sphaeria pinodes* *Sphaerella pinodes* *Mycosphaerella pinodes* *Didymellina pinodes* *Ascochyta pinodes* *Peyronellaea pinodes*	–

^1^ Simplified species name used throughout this review, following the currently preferred denomination in the EPPO Global Database [29,30,31,32]. ^2^ Name stated as “Current” in MycoBank Database [33]. ^3^ Same-type synonym (homotypic synonym). ^4^ Different-type synonym (heterotypic synonym).

**Table 2:** *Pisum* germplasm with reported partial resistance to Ascochyta blight pathogens.

Pathogen	Experimental Conditions ^1^	Accession Number	Current Name	Taxon	Reported Resistance ^2^	Scale ^3^	Ref. ^4^
*A. pisi* (race C)	CCs (seedling)	−	Rondo	*P. sativum*	1	DR 0–3	[81]
		−	Nina	*P. sativum*	1		
		−	Madonna	*P. sativum*	1		
*A. pisi* (race II)	CCs (seedling)	−	Ottawa A-100	*P. sativum*	A-1	DR Leaves: A–E Stems: 1–5 (according to [82])	[69]
*A. pisi* (races I, III)	CCs (seedling)	−	Century (Creamette)	*P. sativum*	“Moderately resistant”	DR Leaves: A–E Stems: 1–5 (according to [82])	[70]
*A. koolunga*	CCs (seedling)	ATC 866	−	*P. sativum*	Leaflets: 155.9	AUDPC Overall leaflets 344; Overall stem 410	[80]
		ATC 864	−	*P. sativum*	Stem: 114.4		
		ATC1490	−	*P. sativum*	0.19	DR relative to Kaspa	[57]
		ATC1667	−	*P. sativum*	0.21		
*D. pinodella*	CCs (seedling)	−	Kaspa	*P. sativum*	Leaflets: 136.8	AUDPC Overall leaflets 344; Overall stem 265	[80]
		ATC 866	−	*P. sativum*	Stem: 64.3		
		−	10HP249-11HO-7	*P. sativum*	1	DR 0–9 (according to [72])	[58]
		−	OZP1305	*P. sativum*	1		
		−	ID89-1	*P. sativum*	1.3	DR Foot rot 0–5	[35]
		−	WA110-42	*P. sativum*	1.3		
		PI 429348	−	*P. sativum*	1.3		
		PI 429349	−	*P. sativum*	1.3		
		−	74SN3	*P. sativum*	1.3		
		−	Fenn	*P. sativum*	1.3		
		PI 429349	−	*P. sativum*	1.3		
		−	74SN3	*P. sativum*	1.3		
*D. pinodes*	Field	JI 1006	−	*P. fulvum*	Strong early resistance	Not reported	[83]
		JI 1012	−	*P. fulvum*	Strong early resistance	Not reported	[75]
		PI 142441	−	*P. sativum*	25.0%	% DS Check Radley 24%	[49]
		PI 142442	−	*P. sativum*	22.5%		
		PI 381132	Prusian Blue	*P. sativum*	26.5%		
		PI 404221	Melkosemiannyj 2	*P. sativum*	24.0%		
		PI 413691	−	*P. sativum*	25.5%		
	Field (Artificially inoculated)	−	Radley	*P. sativum*	Leaves: 22.5%; Stems: 5.4%; Pods: 4.8%; Seeds: 19%	% DS	[72]
		−	Carneval	*P. sativum*	Pods: 3%		
		PI 601513	Danto	*P. sativum*	Pods: 6.5%		
		−	Majoret	*P. sativum*	Seeds: 15.5%		
		−	Miko	*P. sativum*	Seeds: 12%		
		−	Yellowhead	*P. sativum*	Pods: 2%; Seeds: 13%		
		PI 273605	−	*P. sativum*	Pods: 2%; Seeds: 0.5%		
	CCs (seedlings)	IPIP201831	DP	*P. sativum*	2.6	DR 0–5 (according to [66])	[22]
		IPIP201683	FP	*P. sativum*	2.3		
		−	Melrose	*P. sativum*	2.7		
		−	11HP302-12HO-1	*P. sativum*	2	DR 0–9 (according to [72])	[58]
		−	06P822-(F5)-BSR-3	*P. sativum*	Leaflets: 48.8	AUDPC Overall leaflets 235; Overall stem 244	[80]
		−	Dundale	*P. sativum*	Leaflets: 51.8		
		ATC 2312	−	*P. sativum*	Stem: 68		
		JI 252	−	*P. sativum*	Stem: Resistant	DR 0–5	[53]
		JI 103	−	*P. sativum*	Stem: Resistant		
		JI 190	SA 1160	*P. sativum*	Stem: 1.94, Leaves: 4.71	DR 0–7	[83]
		ATC1498	−	*P. sativum*	0.04	DR relative to Kaspa	[57]
		ATC3759	−	*P. sativum*	0.16		
	CCs (seedlings) + field	−	CDC Striker	*P. sativum*	CCs: 4.6, Field: 1.9	DR 0–5 (according to [66]) Check CDC Striker CCs: 4.6; Field: 1.9	[78]
		IFPI 3232	P651	*P. fulvum*	CCs: 2.8, Field: 1.9		
		JI 1006	−	*P. fulvum*	CCs: 3.2, Field: 1.4		
		PI 595936	−	*P. fulvum*	CCs: 3.2, Field 1.7		
		PI 344538	−	*P. sat. elatius*	CCs: 2.7, Field: 1.6		
		W6 15017	−	*P. fulvum*	CCs: 3.3, Field: 1.0		
		PI 560061	−	*P. fulvum*	CCs: 3.2, Field: 1.0		
		PI 344005	P18	*P. sat. elatius*	CCs: 2.3, Field: 18%	CCs: DR 0–5 (according to [66]) Field: %DS Check Radley CCs: 3.7; Field: 23%	[77]
		IFPI 3232	P651	*P. fulvum*	CCs: 1.2, Field: 5%		
		IFPI 3253	P658	*P. fulvum*	CCs: 2.2, Field: 10%		
		IFPI 3260	P660	*P. fulvum*	CCs: 2.4, Field: 17%		
		IFPI 3261	P661	*P. fulvum*	CCs: 2.4, Field: 10%		
		IFPI 3280	P665	*P. fulvum*	CCs: 2.0, Field: 18%		
		IFPI 3341	P672	*P. sat. elatius*	CCs: 1.6, Field: 19%		
		IFPI 3334	P670	*P. sat. elatius*	CCs: 1.0, Field: 12%		
		JI 1006	−	*P. fulvum*	CCs: 1.8, Field: 5%		
	CCs + field	PI 860323	−	*P. sativum*	CCs: 1.0; Field: 0.2	CCs: DR 0–4 Field: DR 0–9 (according to [72])	[73]
		PI 203069	−	*P. sativum*	CCs: 1.0; Field: 0.9		
		PI 275826	−	*P. sativum*	CCs: 0.8; Field: 1.3		
		PI 269821	William Massey	*P. sativum*	CCs: 1.0; Field: 1.2		
		W6 15287	Marx 272	*P. sativum*	CCs: 1.0; Field: 1.3		
*D. pinodes* + *A. pisi* (mixed)	Field (seedling and adult)	−	Cob-192/75	*P. sativum*	Seedling: R; Adult: I	Susceptible (S)/ /Intermediate (I)/ /Resistant (R)	[71]
		JI 411	Cobri	*P. sativum*	Seedling: R; Adult: I		
		JI 513	Olympic A Sharpes	*P. sativum*	Seedling: R; Adult: I		
		JI 584	Recette-C-4	*P. sativum*	Seedling: R; Adult: I		
		JI 494	Sharpes-20065	*P. sativum*	Seedling: R; Adult: I		
		JI 580	Sun Valley	*P. sativum*	Seedling: R; Adult: I		
		JI 573	−	*P. sativum*	Seedling: R; Adult: I		
		PI 17901	−	*P. sativum*	Seedling: R; Adult: I		
		PI 163131	Matar	*P. sativum*	Seedling: R; Adult: I		
		JI 569	−	*P. sativum*	Seedling: R; Adult: I		
		PI 236493	Lamprecht #375	*P. sativum*	Seedling: R; Adult: I		
		PI 166159	−	*P. sativum*	Seedling: R; Adult: I		

^1^ Experimental setting and developmental stage of the host (when reported) for which the original study states the resistance response (CCs: Controlled conditions; Field: field conditions). ^2^ Resistance level of the genotype according to the original study. ^3^ Resistance scoring variable (DR: Disease Rate; DS: Disease Severity; AUDPC: Area under the disease progression curve) and scale, employed to characterize the resistance level in the original study. When reported, reference values for the check genotype are indicated. ^4^ Bibliographical reference. Where resistance was reported in multiple works, the citation shown corresponds to the study providing the most comprehensive characterization of the response.

**Table 3:** Consensus loci associated with Ascochyta blight resistance in pea.

Locus	LG	Consensus Region ^1^	Associated Marker(s) ^2^	Population/Panel	Experimental Conditions	Ref.
−	LG I	−	SilicoDArT-4661533 (SilicoDArT)	6 RIL populations	CC	[57]
Mb-II	LG II	−	ccta2 (AFLP)	Carneval × MP1401 (RIL)	Field	[99]
MpII.1		−	sut1_SNP1 (SNP)/OPRS4_699 (RAPD)	P665 × Messire (RIL)	Field	[143]
mpIII-1	LG III	MDAF.3.1 (Dp.3.2)	E08-980 (RAPD)	JI296 × DP (RIL)	CCs + field	[111]
MpIII.1			OPW5387 (RAPD); OPM6598 (RAPD)	P665 × Messire (RIL)	Field	[139]
Asc3.2		MDAF.3.1	M3P2-418 (AFLP); J12-1400 (RAPD)	A26 × Rovar (F2-derived)	Field	[110]
MpIII.5		Dp.3.9	agpl1_SNP2 (SNP)/MSU515_SNP3 (SNP)	P665 × Messire (RIL)	CCs	[143]
MpIII.3_DRl_05		MDAF.3.2	AA175 (SSR)	P665 × Messire (RIL)	Field	[141]
MpIII.3_DRst_05			AA175 (SSR)	P665 × Messire (RIL)	Field	[141]
MpIII.3_DS_05			AA175 (SSR)	P665 × Messire (RIL)	Field	[141]
MpIII.3_DRst_06			OPAI14_1273 (RAPD)/OPAI14_1353 (RAPD)	P665 × Messire (RIL)	Field	[141]
MpIII.3_DS_06			AA175 (SSR)	P665 × Messire (RIL)	Field	[141]
Asc3.1			P10-711 (RAPD)	A88 × Rovar (F2-derived)	Field	[51]
mpIII-3		MDAF.3.2 (Dp.3.3)	V03-1000 (RAPD); PSMPSAA175 (SSR)	JI296 × DP (RIL)	CCs + field	[111]
abIII-1			PsC8780p118 (SNP); PsC22609p103 (SNP)	Alfetta × P651 (RIL)	CCs + field	[100]
mpIII-4		−	F09-1900 (RAPD)	JI296 × DP (RIL)	CCs + field	[111]
mpIII-5		−	PSMPSAA374a (SSR)	JI296 × DP (RIL)	Field	[111]
−		−	S5LG3_198269966 (SNP)	GWAS panel	CCs	[103]
Mb-IV	LG IV	−	cccc1 (AFLP)	Carneval × MP1401 (RIL)	Field	[99]
abI-IV-2.1		−	Sc1762_271077 (SNP)	Alfetta × P651 (HIF)	Field	[65]
abI-IV-2.2		−	Sc33287_25420 (SNP)	Alfetta × P651 (HIF)	Field	[65]
MpV.2	LG V	MDAF.5.2	OPM4_490 (RAPD)/OPK6_887 (RAPD)	P665 × Messire (RIL)	CCs	[143]
MpV.3			agpl1_SNP2 (SNP)/MSU515_SNP3 (SNP)	P665 × Messire (RIL)	CCs	[143]
mpVa-1			PSMPSAA163.2 (SSR)	JI296 × DP (RIL)	CCs + field	[111]
Asc5.1		MDAF.5.2 (Dp.5.2–Dp.5.3)	sAFP2P2c (AFLP)	88 × Rovar; A26 × Rovar (F2-derived)	Field	[51]
mpVI-1	LG VI	MDAF.6.2 (Dp.6.2)	G04-950 (RAPD)	JI296 × DP (RIL)	CCs	[111]
Mb-VI		−	acct1 (AFLP)	Carneval × MP1401 (RIL)	Field	[99]
−		−	S1LG6_26051507 (SNP)	GWAS panel	CCs	[103]
−		−	S1LG6_369964198 (SNP)	GWAS panel	CCs	[103]
mpVII-1	LG VII	Dp.7.1	PSMPSAA399 (SSR)	JI296 × DP (RIL)	CCs + field	[111]
Asc7.2		Dp.7.3	S15-1330 (RAPD); M3P8-199 (AFLP)	A26 × Rovar (F2-derived)	Field	[110]
mpVII-2			Z17-550 (RAPD)	JI296 × DP (RIL)	Field	[111]
−		−	S7LG7_336950420 (SNP)	GWAS panel	CCs	[103]
−		−	S7LG7_37540311 (SNP)	GWAS panel	CCs	[103]

^1^ Meta disease architecture frost (MDAF) designations follow the meta-analysis by Boutet et al. [101]. ^2^ Peak marker(s) reported for the QTL, or the closest marker(s) on the original linkage map. When multiple marker names are listed, “/” is used to denote flanking pairs (left/right) and “;” to separate additional, non-paired markers of equal relevance. The marker type is specified in parentheses after the marker name.

## Data Availability

No new data were created or analysed in this study.

## Abbreviations

The following abbreviations are used in this manuscript:

MAS	marker-assisted selection
GS	genomic selection
SDHIs	succinate dehydrogenase inhibitors
DMIs	demethylation inhibitors
QoIs	quinone outside inhibitors
MoA	mode of action
EC_50_	half maximal effective concentration
USDA	United States Department of Agriculture
VIR	N.I. Vavilov All-Russian Institute of Plant Genetic Resources
ICARDA	International Center for Agricultural Research in the Dry Areas
CCs	controlled conditions
DR	disease rate
DS	disease severity
AUDPC	area under the disease progress curve
CPVO	Community Plant Variety Office
ROS	reactive oxygen species
PR	pathogenesis-related
QTL/QTLs	quantitative trait locus/loci
RAPD	random amplified polymorphic DNA
RFLP	restriction fragment length polymorphism
AFLP	amplified fragment length polymorphism
STS	sequence-tagged site
RIL	recombinant inbred line
SSR	simple sequence repeat
SNP	single-nucleotide polymorphism
GWAS	heterogeneous inbred family
MTA	marker–trait association
DArT	Diversity Arrays Technology
MDAF	meta disease architecture frost
RGA	resistance gene analogue
MAP	mitogen-activated protein
CAZyme	carbohydrate-active enzyme
DEGs	differentially expressed genes
ABA	abscisic acid
RNA-seq	RNA sequencing
BSR-Seq	bulk segregant RNA sequencing
2-DE	two-dimensional electrophoresis
LEA	late embryogenesis abundant
LC-MS/MS	liquid chromatography–tandem mass spectroscopy
LC-MS	liquid chromatography–mass spectroscopy
G×E	genotype-by-environment
GBS	genotyping-by-sequencing
